# Draft Genome Sequence of Bacillus velezensis PEBA20, a Strain with a Plant Growth-Promoting Effect and Biocontrol Potential

**DOI:** 10.1128/genomeA.00286-18

**Published:** 2018-05-24

**Authors:** Wei-Jian Kong, Yong-Cai Yan, Xiang-Ying Li, Zhen-Yu Liu

**Affiliations:** a College of Plant Protection, Shandong Agricultural University, Shandong Provincial Key Laboratory of Agricultural Microbiology, Tai’an, Shandong Province, People’s Republic of China

## Abstract

Bacillus velezensis PEBA20 is a poplar endophyte with biocontrol activities and plant growth-promoting effects. The genome of B. velezensis PEBA20 was sequenced and the draft genome assembled, with a length of 4,249,176 bp and 4,487 genes.

## GENOME ANNOUNCEMENT

Bacillus spp. can produce a variety of antimicrobial substances, with most of them being low-molecular-weight antimicrobial peptides and some protein antagonists. Bacillus velezensis PEBA20 showed antibacterial effects against a variety of bacterial and fungal plant diseases, and it showed plant growth-promoting effects (1, 2). The genome of PEBA20 was sequenced using the Illumina HiSeq 2500 platform and tested for quality by FastQC. Genome assembly was performed using the RS_AHA_Scaffolding.1 program of smrtanalysis-2.1.1. Subsystem functional classification was predicted using the Rapid Annotations using Subsystems Technology (RAST) server (3). Analysis of antibiotic properties was conducted by a local BLAST comparison using the Antibiotic Resistance Genes Database (ARDB) (4). In the draft genome of B. velezensis PEBA20, a length of 4,249,176 bp is reported, and a total of 4,487 genes were predicted, of which 4,304 are protein-coding genes and 183 are RNAs.

The genome of B. velezensis PEBA20 comprises several genes related to plant growth promotion properties, such as genes encoding indole-3-acetic acid acetyltransferase, indole-3-acetaldehyde dehydrogenase, and nitrilase, which contribute to the biogenesis of indole-3-acetic acid (IAA). Bacillus spp. can produce a variety of antimicrobial substances through the ribosomal synthesis pathway and nonribosomal synthesis pathway (5, 6). The B. velezensis PEBA20 genome contains 10 gene clusters, including ribosomally synthesized peptides, nonribosomal peptide synthetases, and polyketide synthases. The mersacidin synthesis gene cluster exists in the B. velezensis PEBA20 genome. In a comparison with the B. velezensis B9601-Y2 genome, PEBA20 contains the complete gene cluster *mrsK2*, *mrsR2*, *mrsF*, *mrsG*, *mrsE*, *mrsA*, *mrsR1*, *mrsM*, and *mrsT*, which is the same as B. velezensis B9601-Y2; therefore, PEBA20 can synthesize mersacidin the same as B9601-Y2 and B. velezensis BH072 (7, 8). However, B. velezensis FZB42 lacks *mrsA*, *mrsR1*, *mrsD*, *mrsM*, and *mrsT*, though it has an *mrs* gene cluster; therefore, it is unable to synthesize mersacidin (6, 9). We found the presence of an amylocyclicin synthesis gene cluster in the genome of B. velezensis PEBA20, consisting of 6 genes with a total length of 4,490 bp; these characteristics were the same as those for B. velezensis FZB42 (10). The synthesis of bacillomycin D, fengycin, and surfactin was regulated by three operons, *bamD*, *fen*, and *srf*, and it also relies on the 4ʹ-phosphopantetheine transferase gene (*sfp*) to transfer coenzyme A (CoA) to serine residues to activate peptide carrier proteins (PCPs). *bamD*, *fen*, *srf*, and *sfp* were found in the genome of B. velezensis PEBA20. B. subtilis 168 is unable to synthesize surfactant because it lacks the *sfp* gene. B. velezensis B9601-Y2 lacks of the 3' end of *srfA* and the 5' end of *srfB*, so it cannot also synthesize surfactants (11). There are three polyketide synthesis gene clusters in the B. velezensis PEBA20 genome, *dfn*, *mln*, and *bae*, which contribute to the synthesis of difficidin, macrolactin, and bacillaene, respectively. The gene cluster *bae* also exists in B. velezensis B9601-Y2, B. velezensis FZB42, and B. subtilis 168 (12). However, B. subtilis 168 cannot synthesize bacillaene, which may be related to the mutation of *sfp* (11, 12).

### Accession number(s).

This whole-genome shotgun project has been deposited in NCBI/GenBank under the accession number PVHM00000000. The version described in this paper is version PVHM01000000.
